# The use of oral and enteral tube‐fed arginine supplementation in pressure injury care: A systematic review and meta‐analysis

**DOI:** 10.1002/nop2.974

**Published:** 2021-06-25

**Authors:** Sahar Cheshmeh, Niloofar Hojati, Arman Mohammadi, Negin Rahmani, Shima Moradi, Yahya Pasdar, Negin Elahi

**Affiliations:** ^1^ School of Nutritional Sciences and Food Technology Kermanshah University of Medical Sciences Kermanshah Iran; ^2^ Department of Pediatric School of Nursing and Midwifery Shahid Beheshti University of Medical Sciences Tehran Iran; ^3^ Julius Maximillian University of Wuerzburg Wuerzburg Germany; ^4^ Department of Nutritional Sciences Research Center for Environmental Determinants of Health (RCEDH) Health Institute Kermanshah University of Medical Sciences Kermanshah Iran

**Keywords:** arginine, meta‐analysis, pressure injury, pressure ulcer, systematic review

## Abstract

**Aim:**

Pressure injuries (PIs) are one of the most common complications related to immobility, especially in hospitalized patients, which lead to increased morbidity, infection and overall decreased quality of life. Arginine supplementation may prevent the development of PIs. This study has summarized the findings of studies on the effect of arginine supplementation on PI healing.

**Design:**

Systematic review and meta‐analysis.

**Methods:**

This study was conducted on online electronic databases including PubMed, Scopus, Web of Science, Google Scholar and Embase to identify relevant clinical trial studies up to September 2020. The pooled effect size of arginine supplement effects on PI was evaluated with standard mean difference (SMD) with 95% confidence interval (CI).

**Results:**

Eight studies met the inclusion criteria for this meta‐analysis with 196 patients. PIs were significantly improved with Arginine supplementation (SMD: −0.6; CI 95%: −0.9 to −0.3, *I^2^*: 72.5%, *p* = .001). Subgroup analysis showed that administering Arginine supplement more than 15 g/day had more beneficial effects on the healing of PIs (SMD: −2.8; CI 95%: −4.08 to −1.52, *I^2^*: 54.7%, *p* = .138).

**Conclusions:**

Our findings suggest that the administration of Arginine supplement in patients with PIs can accelerate the healing of this type of ulcer. Arginine is a supplement, and primary treatment is still needed to optimize PI healing. Therefore, arginine supplementation in addition to primary treatment seems to be an appropriate approach for the healing of PIs. Further well‐designed studies are necessary to prevent the development of PIs compared to their primary treatment.

## 1 INTRODUCTION

Pressure ulcers—formerly called bed sores—affect a particular area of the body and cause damage to the skin and underlying tissues (Jomar et al., 2019). In the new definition of pressure ulcer based on the National Pressure Ulcer Advisory Panel (NPUAP), the term pressure injuries (PIs) are used instead and refer to the usual damage to body tissues due to pressure caused by the protrusion of bones or medical device or any other device (Edsberg et al., 2016). The PIs are associated with painful, costly, often preventable complications that many people in hospitals, nursing homes and home care are prone to (Beeckman et al., 2011). Despite the increasing costs of preventing the PIs and significant nursing care, the PIs are still a major health problem (Dalvand et al., 2018; Etafa et al., 2018). On the other hand, proper nutritional interventions have been found to be appropriate in healing these ulcers (Cereda et al., 2017; Yamanaka et al., 2017). Therefore, reviewing studies focusing on nutritional interventions can be helpful in healing these ulcers for nursing care.

## 2 BACKGROUND

PIs are usually located in areas where the bones of the body are and are caused either by medical devices or by long‐term positioning as a result of pressure alone or with shear (force?) leading to damaged skin or body tissue (Jomar et al., 2019). They are often unavoidable consequences of a medical condition which can impose a huge burden on healthcare systems (Mervis & Phillips, 2019b). Results from a systematic review and meta‐analysis by Li et al. (Li et al., 2020) showed that global incidence rate of hospital PIs in adults was 8.5% (CI 95:7.6–9.3). In general, PIs are common in 5%–15% of hospitalized patients, which is more common in intensive care units than the other wards. On the other hand, the prevalence of these ulcers is higher in hospitalized patients than in nursing homes and homes care (Mervis & Phillips, 2019a).

PIs are associated with decreased mobility and quality of life via increasing pain, infection, prolonged hospitalization and decreasing physical function, as well as resulting in both morbidity and mortality (Ledger et al., 2020; Li et al., 2020). Despite preventive strategies, they are still one of the most common complications, especially in a hospital intensive care unit (Mervis & Phillips, 2019b). Key primary therapeutic interventions for PIs are aimed at distributing pressure between the skin and the surface in contact with it, as well as wound dressings (Atkinson & Cullum, 2018). There are various methods for the treatment and prevention of PIs. Among them, researchers have found that proper nutrition and the use of nutritional supplements can heal these wounds (Sugihara et al., 2018; van Anholt et al., 2010; Yamanaka et al., 2017).

Some studies have shown that the use of oral and enteral tube‐fed arginine supplementation is effective in healing PIs and can prevent their development (Bauer et al., 2013; Brewer et al., 2010; Cereda et al., 2015; Leigh et al., 2012; Yamanaka et al., 2017). Recently, Cereda et al. (Cereda, Neyens, et al., 2017) showed that formula enriched with Arginine could improve PIs in hospitalized patients. Arginine is a semi‐essential amino acid that is a precursor of proteins such as collagen and improves the function of T lymphocytes, enhances cell proliferation and regulates nitrogen balance (Cereda et al., 2015). However, another meta‐analysis found no evidence that oral and tube‐fed supplementation has positive effects on wound healing (Langer & Fink, 2014). Since these reviews were from several years ago, the number of studies analysed in this area was limited, and the effect of arginine supplementation on PIs alone was not evaluated. In addition, the findings of these studies were contradictory. Since PIs are an unavoidable complication in immobilized patients, detecting effective nutritional factors can be an appropriate strategy to reduce the chance of their development. Therefore, according to the contradictory findings of previous studies and the fact that one study did not consider the effect of arginine alone on PIs, the present systematic review and meta‐analysis study aimed to summarize the findings of studies on the effect of oral and tube‐fed supplementation with arginine on the healing of PIs.

## 3 METHODOLOGY

### 3.1 Design

We used a systematic review and meta‐analysis study design by updated data on patients from all available interventional studies based on the Preferred Reporting Items for Systematic Reviews and Meta‐Analyses (PRISMA) guideline (Moher et al., 2015).

### 3.2 Methods

#### 3.2.1 Search strategy

Literature search was conducted using online electronic databases including PubMed, Scopus, Web of Science, Google Scholar and Embase to identify relevant interventional studies in which the effects of Arginine supplementation on the healing of PIs were investigated. The following search‐related Medical subject headings (MeSH) and non‐MeSH keywords were used by two independent researchers: Arginine OR L‐Arginine AND Pressure Ulcer OR Bedsore OR Decubitus Ulcer. The articles up to September 2020 were selected.

In addition, we screened the relevant references of review studies to find articles that we may not have found. In this systematic search, limitations in the location of study, race, publication time and language were not considered.

#### 3.2.2 Inclusion and exclusion criteria

**FIGURE 1:**
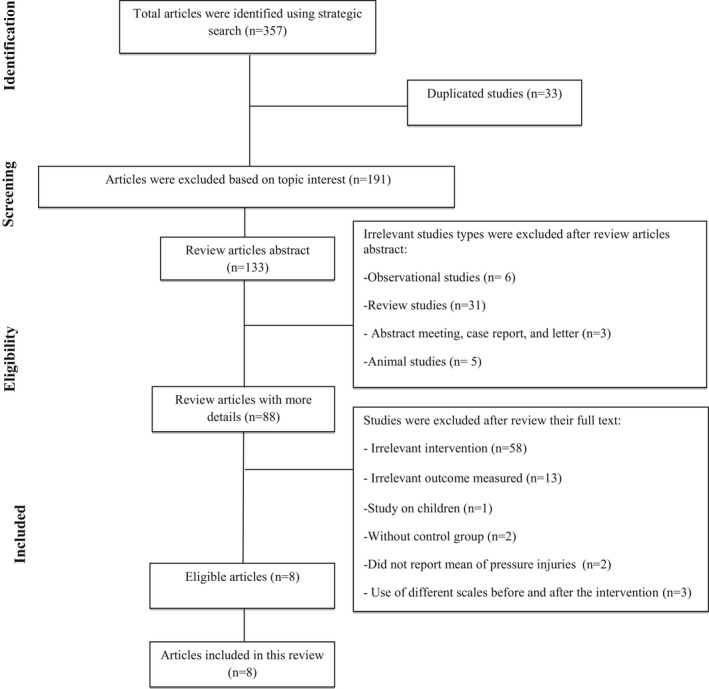
Flow chart of studies selection

The following studies were included in this systematic review: (1) any study with clinical trial design that was conducted on adults with PI; (2) case groups in these studies received arginine supplementation and the effect on the improvement of PI was assessed; (3) studies that reported mean ± standard deviation (*SD*) of PIs before and after intervention; and (4) studies that had a control group. Overall, 357 articles were identified in the initial systematic search. After removing 33 duplicated studies, 324 articles were screened based on the interest topic and 191 irrelevant articles were excluded. In addition, 45 irrelevant studies were excluded after reviewing the articles’ abstracts. In addition, we did not include one study conducted on paediatric subjects (Masumoto et al., 2011). After that, 88 articles were reviewed with more detail and we excluded 59 articles that did not administer arginine supplementation and 13 articles that did not assess the introduced outcome. Furthermore, we excluded articles that did not have control groups (*n* = 2) (Heyman et al., 2008; Yatabe et al., 2011) and did not report the mean of PIs (*n* = 2) (Frias Soriano et al., 2004; Hommel et al., 2007). Moreover, we excluded three studies that used different scales before and after the intervention (*n* = 3) (Brewer et al., 2010; Cereda et al., 2015; Houwing et al., 2003). These studies reported PI areas based on cm^2^ or mm^2^, used mean or median depending on the normality of their data and did not use scales such as PUSH; therefore, they were not convertible in the analysis and we had to remove them. Overall, 8 articles were eligible for this systematic review and meta‐analysis. (Figure 1).

#### 3.2.3 Quality assessment

We applied Critical Appraisal Skills Program (CASP) checklist to assess the quality of included articles ((CASP) and http://www.casp‐uk.net/checklists). Risk of bias was assessed using the CASP for randomized clinical trial (Available from: http://www.casp‐uk.net/casp‐tools‐checklists) by two independent researchers. We resolved any disagreements by discussion. This checklist has three sections as follows: validity, results and clinical relevance, and 11 questions are asked in total. The responses were either Yes, Can't Tell or No. The answers Yes, Can't Tell, and No were considered low risk, unsure risk and high risk, respectively. (Table 1) Publication bias among these included studies was assessed by funnel plots as well as Egger's regression and Begg's tests.

**TABLE 1:** Quality assessment of included articles using CASP checklist

CASP Checklist/Frist name, year	Benati et al., 2001	Desneves et al., 2005	Cereda et al., 2009	van Anholt et al., 2010	Leigh et al., 2012	Bauer et al., 2013	Wong et al., 2014	Yamanaka et al., 2017
Did the trial address a clearly focused issue?	Yes	yes	yes	yes	yes	yes	yes	yes
Was the assignment of patients to treatments randomized?	Yes	yes	yes	yes	yes	yes	yes	yes
Were all of the patients who entered the trial properly accounted for at its conclusion?	Yes	yes	yes	yes	yes	yes	yes	yes
Were patients, health workers and study personnel ‘blind’ to treatment?	Can't Tell	yes	yes	yes	yes	yes	yes	no
Were the groups similar at the start of the trial?	Yes	Yes	yes	yes	yes	yes	yes	yes
Aside from the experimental intervention, were the groups treated equally?	Yes	yes	yes	yes	yes	yes	yes	yes
How large was the treatment effect?	Yes	yes	yes	yes	yes	yes	yes	yes
How precise was the estimate of the treatment effect?	Yes	yes	yes	yes	yes	yes	yes	yes
Can the results be applied to the local population, or in your context?	Yes	yes	yes	yes	yes	yes	yes	no
Were all clinically important outcomes considered?	Yes	yes	yes	yes	yes	yes	yes	yes
Are the benefits worth the harms and costs?	No	yes	yes	yes	yes	yes	yes	yes

#### 3.2.4 Data extraction

Data were extracted independently by two researchers using a standardized data collection checklist and any disagreements were discussed and resolved. This checklist included the name of the first author of each study, year of study, study sample size, disease type, dose of arginine, type of supplement, duration of intervention and PIs measuring tools. The key outcome variable was the mean of PI before and after intervention. This pre‐specified data extraction checklist was designed in Excel (Microsoft Corporation).

#### 3.2.5 Outcome assessment

The reviewed studies measured PIs by Pressure Ulcer Scale for Healing (PUSH) (Bauer et al., 2013; Cereda et al., 2009; Desneves et al., 2005; Leigh et al., 2012; van Anholt et al., 2010; Wong et al., 2014), pressure sore status tool (PSST) (Benati et al., 2001) and DESIGN‐R (Yamanaka et al., 2017). The PUSH tool evaluates wound length and depth, exudate amount, and tissue type that is developed by the National Pressure Ulcer Advisory Panel in 1997 (http://www.npuap.org). Total PUSH score ranged between 0 (completely healed)–17 (severe wound) (3.).

PSST was developed by Bates‐Jensen et al. Bates‐Jensen et al., 1992) in 1992 and includes 13 items including size, depth, edges, undermining, necrotic tissue type and amount, exudate type and amount, skin colour surrounding wound, peripheral tissue oedema and induration, granulation tissue and epithelialization. This score ranges between 13–65 and higher scores are associated with worse wound conditions.

DESIGN‐R evaluates the severity of PIs and monitors their healing. It includes 7 items including depth, exudate, size, inflammation/infection, granulation, necrosis and pocket formation. This scale was introduced by the Scientific Education Committee of the Japanese Society of Pressure Ulcers in 2002 and was revised in 2008 (Ulcers.). Total score ranged from 0 (best condition)–66 (greatest severity).

### 3.3 Statistical analysis

Statistical analysis was performed using STATA, version 14 (Stata Corp, College Station, TX). The pooled effect size of arginine supplement on PI was evaluated with standard mean difference (SMD) with 95% confidence interval (CI). Heterogeneity between these studies was detected by I square (*I^2^*) test. Between‐study heterogeneity was defined as *I^2^* = 50% or more. To find the probable source of heterogeneity, we considered subgroup analysis. Furthermore, sensitivity analysis was applied to find the dependent pooled effect size from each study.

## 4 RESULTS

Our literature search identified eight articles with their characteristics presented in Table 2. All reviewed studies were published between 2001–2017 with 196 included patients. There were 97 patients in the intervention group and 99 patients in the control group. These studies were conducted in Italy (Benati et al., 2001; Cereda et al., 2009), Australia (Bauer et al., 2013; Desneves et al., 2005; Leigh et al., 2012), the Netherlands (van Anholt et al., 2010), Singapore (Wong et al., 2014) and Japan (Yamanaka et al., 2017). Dose of arginine supplement varied from 2.5–18 gr and the duration of intervention varied from 2–12 week. Six studies assessed PIs using PUSH score (Bauer et al., 2013; Cereda et al., 2009; Desneves et al., 2005; Leigh et al., 2012; van Anholt et al., 2010; Wong et al., 2014), ones used PSST (Benati et al., 2001) and the other DESIGN‐R scores (Yamanaka et al., 2017). Result of funnel plot showed that there was no publication bias among these reviewed studies. In addition, results of Egger's regression (*p* = .108) and Begg's test (*p* = .051) were not significant. Sensitivity analysis of the studies showed that there was no change in the overall result with the elimination of each study.

**TABLE 2:** Characteristics of reviewed studies

First Author, year	Country, Sample size (men/women)	Case/control	Age (year)	Dose of Arginine (gr)	Supplement type	Duration (week)	Pressure ulcer tools	Intervention group		Placebo group	
								Baseline	Final	Baseline	Final
Benati G, 2001 (Benati et al., 2001)	Italy, 11 (6 men/5 women)	6/5	72–91	15	High protein calorie	2	PSST	45.4 ± 15.1	21.7 ± 7.2	42.7 ± 12.5	45.1 ± 7.2
Katherine J, 2005 (Desneves et al., 2005)	Australia, 11 (7 men/4 women)	5/6	37–92	18	High protein calorie	3	PUSH	9.4 ± 1.2	2.6 ± 0.6	8.7 ± 1	7 ± 1.5
Cereda E, 2009 (Cereda et al., 2009)	Italy, 28 (10 men/18 women)	13/15	≥65	12	Enteral formula	12	PUSH	13.5 ± 2.2	7.4 ± 3.4	14 ± 2.6	10.7 ± 3.4
van Anholt R, 2010 (van Anholt et al., 2010)	Netherlands, 43 (19 men/24 women)	22/21	18–70	9	Nutritional supplement	8	PUSH	11.5 ± 0.7	4.9 ± 0.9	11.4 ± 0.7	5.5 ± 1
Leigh B, 2012 (Leigh et al., 2012)	Australia, 23 (14 men/9 women)	11/12	≥60	9	Nutritional supplement	3	PUSH	8.1 ± 1	4.8 ± 1.6	8.9 ± 0.7	5.5 ± 1.3
J. D. Bauer, 2013 (Bauer et al., 2013)	Australia, 24 (11 men/13 women)	12/12	≥18	9	Wound‐specific supplement	8	PUSH	13.9 ± 0.7	−0.6^a^	14.2 ± 1.2	−4.8
A. Wong, 2014 (Wong et al., 2014)	Singapore, 23 (9 men/14 women)	11/12	≥21	14	Oral nutritional supplements	2	PUSH	12.4 ± 0.7	9.6 ± 1.1	12.4 ± 0.7	10.6 ± 1.1
Yamanaka H, 2017 (Yamanaka et al., 2017)	Japan, 33 (12 men/21 women)	17/16	≥20	2.5	Supplement drink	4	DESIGN‐R	14.1 ± 5.6	11.5 ± 12.9	15.9 ± 5.7	13.9 ± 7.9

Abbreviations: PSST, Pressure sore status tool; PUSH, Pressure Ulcer Scale for Healing.

^a^ Mean of changes.

**FIGURE 2:**
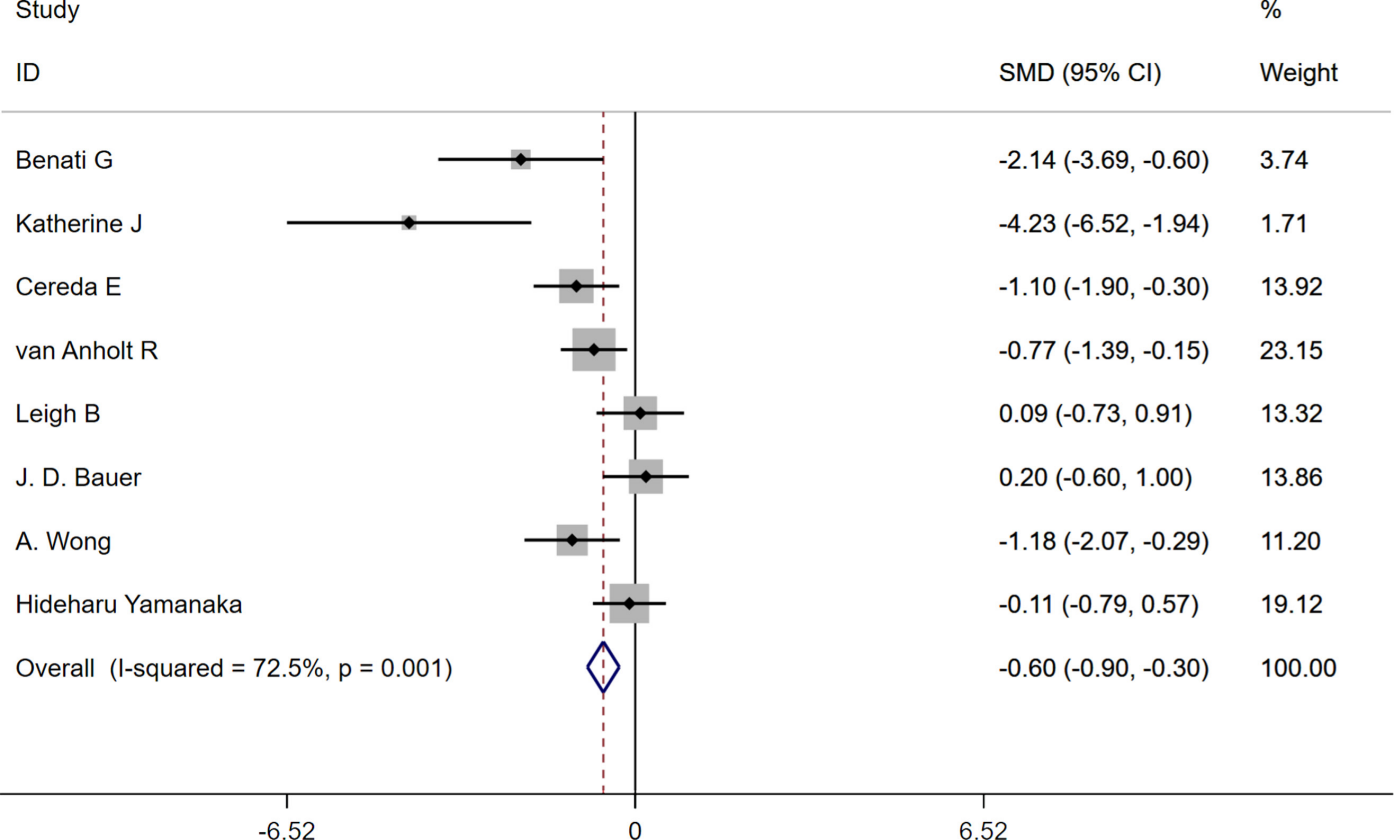
Forest plot for the effect of arginine supplementation on pressure injuries

In the current review study, we observed that arginine supplementation significantly improved PIs (SMD: −0.6; CI 95%: −0.9 to −0.3). However, there was heterogeneity among the studies (*I^2^*: 72.5%, *p* = .001) (Figure 2). Therefore, we classified these studies according to the administered dose and reanalysed.

Subgroup analysis according to the administered doses showed that administering arginine supplement more than 15 g/day had more effects on PIs (SMD: −2.8; CI 95%: −4.08 to −1.52), (*I^2^*: 54.7%, *p* = .138) compared to arginine supplementation at a dose of less than 15 g/ day (SMD: −0.47; CI 95%: −0.78 to −0.16). However, both of the two doses of arginine supplement significantly improved PIs (*I^2^*: 55.8%, *p* = .046) (Table 3).

**TABLE 3:** Subgroup analysis according to the administered dose of arginine supplementation and effects on pressure ulcer

Variables	Dose of arginine supplementation	
	<15 g/day	≥15/day
No. of included studies	6	2
SMD (CI 95%)	−0.47 (−0.78 to −0.16)	−2.8 (−4.08 to −1.52)
*p*‐value	0.003	<0.001
*I^2^*	55.8%	54.7%
*p*‐heterogeneity	0.046	0.138

Abbreviations: CI, Confidence interval; SMD, Standard mean difference.

## 5 DISCUSSION

Our results highlighted that arginine supplementation significantly improved PIs. In addition, subgroup analysis showed that the administration of higher doses of arginine supplement had a greater effect on reducing the probability of PI development. In the included studies, supplementation with arginine for at least 2 weeks significantly reduced scores of PUSH and PSST. To the best of our knowledge, we conducted this systematic literature review and meta‐analysis on the effects of arginine supplementation on the healing of PI. PIs describe the patient's safety status and the quality of care that is prevalent in patients admitted to intensive care unit (Akhkand et al., 2020). Results from a systematic review on 36 studies showed that the health care cost of PIs in one year amounted to 15,400 USD (Chan et al., 2017). The most important strategy in ulcer healing is the distribution of pressure between the body surface and the objects in contact with body surface to prevent injury. In addition, the injury levels of the body should be bandaged regularly (Atkinson & Cullum, 2018). However, factors contributing to PI development are old age, diabetes, immobility, smoking and poor nutritional status (Wood et al., 2019; Zarei et al., 2019). Low weight or obesity, skin friction and shear stress are other factors involved in PI development which interfere with primary care and other supplementation (Langer & Fink, 2014).

Studies have shown that arginine supplementation can help protein synthesis, improve immune function, division and cell differentiation (Cereda et al., 2015; Schneider & Yahia, 2019). In the current meta‐analysis, PIs was significantly improved in six included studies (Benati et al., 2001; Cereda et al., 2009; Desneves et al., 2005; Leigh et al., 2012; van Anholt et al., 2010; Wong et al., 2014) after Arginine supplementation except studies by J. D. Bauer et al. (Bauer et al., 2013) and Yamanaka H et al. (Yamanaka et al., 2017) compared to control groups. In a study by J. D. Bauer et al. (Bauer et al., 2013), it was observed that the oral standard supplement can improve PI status with the mean change of – 4.8 (CI%: −9.5–0.1) compared to the control group. Yamanaka H et al. (Yamanaka et al., 2017) did not observe any significant difference in PI healing before and after intervention with arginine supplement drink (*p* = .093). Overall, we concluded that PIs were healed after the administration of arginine supplementation. Schneider et al. (Sugihara et al., 2018) reported in a systematic review on elderly patients that arginine supplementation might improve PIs. Another study by P. Liu et al. (Liu et al., 2017) showed that arginine‐enriched enteral formula could heal PIs.

Arginine supplementation has anti‐oxidative and anti‐inflammatory effects in the body (Liang et al., 2018). Increase in reactive oxygen species production has been suggested as an important factor in non‐healing of chronic ulcers (Cano Sanchez et al., 2018). Arginine supplementation by stimulating nitric oxide synthesis affects glutathione metabolism, activates nuclear factor erythroid‐2‐related factor‐2 (Nrf2) pathway and leads to the release of zinc and increased plasma zinc (Bergeron & Guay, 2019). Nrf2 induces antioxidant defence genes in response to oxidative stress and pro‐inflammatory factors (Li et al., 2019). In addition, arginine is also a biological precursor of nitric oxide and can help heal ulcers through the production of nitric oxide (Schneider & Yahia, 2019) and collagen synthesis (Liu et al., 2017). Therefore, arginine supplementation can improve ulcers via the production of nitric oxide and modulating inflammatory and oxidative responses.

### 5.1 Limitations

This study was the first to summarize the findings of previous studies on arginine supplementation and PIs. However, it has several limitations. We could not include articles that had no control group, did not report the mean of PIs, and those that used different scales before and after the intervention. Therefore, only eight articles were eligible for this study and this has led to the limited number of included studies. Other limitations were related to the short duration of intervention and low sample size of the included studies. Furthermore, the different doses used in the included studies were another limitation of our study.

## 6 CONCLUSION

The findings of this meta‐analysis reflect that the arginine supplementation can improve PIs healing. In addition, using higher doses of arginine supplement had greater beneficial effects on the PIs healing. Although nurses are not solely responsible for preventing pressure ulcers, medical teamwork collaboration have a significant impact on PIs protection. However, we cannot generalize the findings of this study to all patients with pressure ulcers because of the small sample size and the length of the interventions. Therefore, further well‐designed studies are needed to shed light on the prevention of PI development and other ulcers.

## CONFLICT OF INTEREST

On behalf of all co‐ authors, the corresponding author states that there is no conflict of interest.

## AUTHOR CONTRIBUTIONS

Study conception and design: Sahar Cheshmeh, Shima Moradi and Arman Mohammadi. Data collection: Sahar Cheshmeh, Niloofar Hojati, Arman Mohammadi and Negin Elahi. Data analysis and interpretation: Sahar Cheshmeh, Shima Moradi and Yahya Pasdar. Drafting of the article: Sahar Cheshmeh, Shima Moradi and Arman Mohammadi. Critical revision of the article: Sahar Cheshmeh, Shima Moradi, Negin Rahmani and Yahya Pasdar. All authors are in agreement with the manuscript and declare that the content has been not published elsewhere, as well as, they agreed to the proposal new authorship shown in this revised manuscript.

## ETHICAL APPROVAL

No ethical approval was necessary for this review manuscript.

## INFORMED CONSENT

No informed consent was necessary for this study as no human subjects were enrolled.

## Data Availability

Data will be available upon request from the corresponding author.
